# NDRG2 Protects the Brain from Excitotoxicity by Facilitating Interstitial Glutamate Uptake

**DOI:** 10.1007/s12975-019-00708-9

**Published:** 2019-06-27

**Authors:** Anqi Yin, Hang Guo, Liang Tao, Guohong Cai, Yazhou Wang, Libo Yao, Lize Xiong, Jian Zhang, Yan Li

**Affiliations:** 1 Department of Anesthesiology and Perioperative Medicine, Xijing Hospital, The Fourth Military Medical University, Xi’an, 710032 Shaanxi China; 2 Department of Anesthesiology, PLA Army General Hospital, Beijing, 100700 China; 3 Institute of Neuroscience, The Fourth Military Medical University, Xi’an, 710032 Shaanxi China; 4 Department of Biochemistry and Molecular Biology, The Fourth Military Medical University, 169 Changle West Road, Xi’an, 710032 Shaanxi China; 5 Department of Anesthesiology, The First Affiliated Hospital of Xi’an Jiaotong University, Xi’an, 710061 Shaanxi China; 6 Center for Brain Science, The First Affiliated Hospital of Xi’an Jiaotong University, 277 Yanta West Road, Xi’an, 710061 Shaanxi China

**Keywords:** N-myc downstream regulated gene 2 (NDRG2), Astrocytes, Glutamate uptake, Excitotoxicity, Brain ischemia

## Abstract

Glutamate is a prominent neurotransmitter responsible for excitatory synaptic transmission and is taken up by sodium-dependent excitatory amino acid transporters (EAATs) on astrocytes to maintain synaptic homeostasis. Here, we report that N-myc downstream regulated gene 2 (NDRG2), a known tumor suppressor, is required to facilitate astroglial glutamate uptake and protect the brain from glutamate excitotoxicity after ischemia. NDRG2 knockout (*Ndrg2*^*−/−*^) mice exhibited an increase in cerebral interstitial glutamate and a reduction in glutamate uptake into astrocytes. The ability of NDRG2 to control EAAT-mediated glutamate uptake into astrocytes required NDRG2 to interact with and promote the function of Na^+^/K^+^-ATPase β1, which could be disrupted by a Na^+^/K^+^-ATPase β1 peptide. The deletion of NDRG2 or treatment with the Na^+^/K^+^-ATPase β1 peptide significantly increased neuronal death upon a glutamate challenge and aggravated brain damage after ischemia. Our findings demonstrate that NDRG2 plays a pivotal role in promoting astroglial glutamate uptake from the interstitial space and protecting the brain from glutamate excitotoxicity.

**Electronic supplementary material:**

The online version of this article (10.1007/s12975-019-00708-9) contains supplementary material, which is available to authorized users.

## Introduction

Glutamate, which is the principal excitatory amino acid, mediates the physiological excitatory synaptic transmission in the brain [1]. Excessive glutamate released from presynaptic neurons induces excitotoxicity, which contributes to the neuropathology observed in acute traumatic and ischemic brain injury, chronic neurodegenerative diseases, and other neurological diseases, such as seizures [2–5].

Rapid glutamate clearance in the synaptic cleft is required for maintaining synaptic homeostasis and preventing excitotoxicity [6–8]. The presynaptic membrane, postsynaptic membrane, and neighboring astrocytes comprise a physical “tripartite synapse” that limits the diffusion of neurotransmitters [9, 10]. Glutamate is predominantly taken up by sodium-dependent transporters known as excitatory amino acid transporters (EAATs), which are mainly localized in tripartite synaptic astrocytes [11, 12]. The glutamate taken up by EAATs is cotransported with Na^+^ into astrocytes [13]. Therefore, the transmembrane Na^+^ gradient in the synaptic space, produced by the export of Na^+^ by Na^+^/K^+^-ATPase, is the essential driving force for glutamate uptake [14, 15].

N-myc downstream regulated gene 2 (NDRG2), a known tumor suppressor protein [16], is primarily expressed in astrocytes rather than in neurons or other glial cells in various brain areas [17–19]. NDRG1, NDRG2, NDRG3, and NDRG4 constitute the NDRG family and are required in cell proliferation and differentiation [20, 21]. *NDRG2* was reported as an early-stage stress response gene, and its expression is upregulated under excitotoxic conditions in the brain, including ischemia [22], hemorrhage [23], trauma [24], and Alzheimer’s disease. To evaluate the potential physiological or pathological roles of NDRG2 in the brain, we generated *NDRG2* knockout (*Ndrg2*^*−/−*^) mice [25]. These mice are susceptible to cerebral ischemia and exhibit increased interstitial glutamate levels in the brain, suggesting that NDRG2 plays an essential role in controlling glutamate excitotoxicity.

Here, we examined this hypothesis and provided evidence that strongly suggests that NDRG2 is required for sodium-dependent glutamate uptake into astrocytes. The NDRG2-mediated astroglial glutamate uptake from the cerebral interstitial fluid is essential for protecting the brain from glutamate excitotoxicity following ischemia.

## Materials and Methods

### Mice

The experimental protocols were reviewed and approved by the Ethics Committee of the Fourth Military Medical University. We made all efforts to minimize the number of mice used and their suffering. The animals were group-housed under a regular 12-h light/dark cycle with access to food and water ad libitum. The *Ndrg2*^*flox/flox*^ mice were crossed with B6.C-Tg(CMV-cre)1Cgn/J mice (Jackson Labs, USA) to generate the *Ndrg2*^*−/−*^ mice. The line was backcrossed to C57BL/6 J more than 20 times. Young adult male (8–10 weeks old) mice were used in this study. All animal procedures were performed in accordance with the guidelines established by the Fourth Military Medical University and with the ARRIVE (Animal Research: Reporting In Vivo Experiments) guidelines.

### Transient Middle Cerebral Artery Occlusion

Mice were anesthetized with 1.5% isoflurane. Body temperature was monitored and maintained at 36.5 to 37.5 °C using a thermostatic pad. The right MCAO surgery was performed as described previously [26], and the cortical blood perfusion was monitored using laser-Doppler flowmetry. A sudden blood flow drop below 15–20% of the baseline value was considered to indicate sufficient occlusion (Fig. S1). After 60 min of occlusion, the mice were reanesthetized to facilitate the removal of blood vessel occlusion for recanalization and reperfusion.

### Immunohistochemistry (IHC) and Immunofluorescence (IF)

Whole mice were fixed with 4% paraformaldehyde via cardiac perfusion. The brain tissues were flash-frozen on dry ice and cut into 12-μm-thick sections using a freezing microtome. First, the brain sections were incubated with the primary antibodies at 4 °C for 12 h. For the immunohistochemistry studies, the sections were incubated with biotinylated secondary antibodies at room temperature for 2 h. The immunostaining was visualized using the streptavidin/peroxidase complex and diaminobenzidine. For the immunofluorescence staining studies, the brain sections were incubated with fluorophore-conjugated secondary antibodies for 2 h at room temperature. The sections then were dyed with 4′,6-diamidino-2-phenylindole (DAPI) for nuclear staining. The staining results were observed and photographed under a laser-scanning confocal microscope. Additional information regarding the antibodies used in this study is provided in the Supplementary material (Table S1).

### Neurological Score Assessment and Cerebral Infarct Volume Measurement

After 60 min of MCAO and 24 h of reperfusion, a neurological score assessment was performed by two observers who were blinded to the study. The following rating scale was used: 0 = no deficit; 1 = failure to extend the right forepaw; 2 = decreased grip strength in the right forepaw; 3 = circling to the right after the tail was pulled; and 4 = spontaneous circling. Then, the mouse brains were removed and cut into coronal slices, and infarct volume was evaluated by 2,3,5-triphenyltetrazolium chloride (TTC) staining as described previously [26]. The edema index was calculated by dividing the right (ipsilateral to the transient middle cerebral artery occlusion (tMCAO)) hemisphere volume by the left (contralateral to the tMCAO) hemisphere volume. The infarct volume was corrected by dividing the infarct volume by the edema index. The infarct volume without the correction for edema was also provided.

### Microdialysis and High-Performance Liquid Chromatography

A microdialysis probe (4-mm length guide cannula, 0.22-mm membrane outer diameter, 1-mm membrane length, MW cutoff 50 kD; Eicom Corp, Tokyo, Japan) was stereotaxically inserted into the right striatum through the cannula guide (2 mm right lateral from the bregma, 0.5 mm anterior, and 4 mm ventral from the dura). Artificial cerebrospinal fluid (ACSF, in mM: NaCl, 124; KCl, 4.4; CaCl_2_, 2; NaHCO_3_, 25; MgSO_4_, 2; KH_2_PO_4_, 1; glucose, 10; pH 7.4) was perfused at a rate of 1 μl/min. The microdialysis samples were continuously collected for 4 h into microvials after 1 h at equilibrium, and these samples were subsequently lyophilized and redissolved in 20 μl of ACSF. The concentrations of glutamate in the microdialysis samples were analyzed by high-performance liquid chromatography (HPLC) as described previously [27]. The concentrations were calculated using LC solution software (Waters, USA) based on the standard samples (Sigma-Aldrich, USA).

### Primary Astrocytic and Neuronal Cultures

Primary astrocytes were obtained from the cerebral cortices of 1- to 3-day-old mouse pups. The brains were minced and trypsinized (0.25% trypsin-EDTA) to produce cell suspensions, which were then plated in poly-L-lysine-coated flasks in Dulbecco’s Modified Eagle’s Medium with 10% fetal bovine serum and maintained at 37 °C and 5% CO_2_. Every 3 days, half of the medium was removed and replaced. When the cells reached confluence after 10 to 14 days, the flasks were shaken at 200 to 220 rpm for 14 to 16 h to remove the microglia and oligodendrocytes. After shaking, the cultures included more than 95% astrocytes as determined by immunofluorescence staining for GFAP. After isolation, the cells were subcultured in different dishes according to distinct protocols.

The neuronal cultures were prepared from the cortices of 18-day-old mouse embryos obtained from pregnant mice by cesarean section. The cerebral cortices were extracted from the embryos and incubated for 30 min in 0.25% trypsin-EDTA. Digested tissues were dissociated by trituration and seeded on poly-L-lysine-coated plates. The culture medium consisted of Neurobasal medium (Invitrogen) supplemented with B27 (Invitrogen). After 2 days, two-thirds of the medium was replaced with fresh medium. The cultures were maintained at 37 °C in a humidified 5% CO_2_ incubator. On the fifth day, the mature neurons were used for the experiments.

### Adeno-Associated Virus Construction and Stereotaxic Injection

NDRG2 adeno-associated virus (AAV) was constructed with the glial fibrillary acidic protein (GfaABC_1_D) promoter that selectively expresses proteins in astrocytes by Genechem Company (Shanghai, China). Intracerebroventricular injection of AAV was carried out using a stereotaxic instrument. Mice were anesthetized with sodium pentobarbital (50 mg/kg, i.p.). A 26-gauge cannula was stereotaxically inserted into the right lateral ventricle (coordinates: 0.22 mm anterior, 1.2 mm right lateral from the bregma, and 2.5 mm ventral from the dura), and 3 μl of AAV (1 × 10^12^ vector genomes/ml) was injected at a constant rate of 1.0 μl/min over 5 min using a micro-syringe pump. The needle was withdrawn slowly over 5 min. Three weeks after AAV injection, mice were subjected to microdialysis or tMCAO.

### Evaluation of Glutamate Uptake

Fluorescein isothiocyanate (FITC) was linked to glutamate. Then, 200 μM FITC-coupled glutamate was added to the primary mice astrocyte cultures. At each time point, the fluorescence intensity in the cultured astrocytes was analyzed. Glutamate uptake was detected using a Live Cell Imaging System (Olympus, Japan).

### Measurement of Intracellular Na^+^

The astrocytes were washed twice with PBS, and 1 μM CoroNa Green indicator (Invitrogen, USA) was then added by dilution from a concentrated stock solution in DMSO. The cells were incubated for 30 min at 37 °C. The loaded cells were washed twice with PBS before the fluorescence was measured. Subsequently, the CoroNa Green indicator was excited at 492 nm, and emission was collected above 516 nm. The images were observed and captured under a laser-scanning confocal microscope (Olympus, Japan).

### Na^+^/K^+^-ATPase Activity Assay

The cellular Na^+^/K^+^-ATPase activity was measured via colorimetric determination using the ATPase Assay Kit (Innova Biosciences). The cells were lysed in deionized water with ultrasonic decomposition. The solubilized protein mixture was centrifuged to remove the cellular debris. The cell suspension (100 μl) was transferred to 2 × 96-well microplates with 100 μl of the substrate/buffer mix and incubated at 37 °C for 15 min. Then, 50 μl of Gold Mix was added to stop the reaction. After 2 min, 20 μl of the stabilizer was added, and the mixture was incubated at room temperature for 30 min. The absorbance was measured using an automated microplate reader at a wavelength of 590 nm. The Na^+^/K^+^-ATPase activity was calculated as the difference between the tested samples (total ATPase activity) and samples assayed in the presence of 2 mM ouabain.

### Immunoblotting Analysis

Proteins were extracted from tissues or cells using a lysis buffer composed of 150 mM NaCl, 50 mM Tris, 0.1% SDS, 1% NP-40, 0.5% sodium deoxycholate, 1 mM EDTA, 1 mM Na_3_VO_4_ and a proteinase inhibitor mixture. The samples were then separated on 10% SDS-PAGE gels, transferred to polyvinylidene difluoride membranes and immunoblotted with the indicated antibodies. The blots were enhanced using a chemiluminescence detection reagent kit (Pierce, USA) and visualized using a Bio-Rad imager and Image Lab software (Bio-Rad, USA). See Supplementary material, Table S1, for more information regarding the antibodies.

### Plasmid Construction and Cell Transfection

The plasmids of Flag-tagged full-length NDRG2, Myc-tagged full-length Na^+^/K^+^-ATPase β1 and its different truncation mutants were constructed by GeneChem (Shanghai, China) by inserting NDRG2 or different Na^+^/K^+^-ATPase β1 fragments into GV140 using XhoI/EcoRI restriction sites. The cells were grown to 80% confluence in 10-cm cell culture dishes before being transiently transfected using Lipofectamine 2000 (Invitrogen, USA) according to the manufacturer’s protocol. The cells were transfected for 48 h at 37 °C before harvesting for the biochemical analyses.

### Coimmunoprecipitation (Co-IP)

The cells were incubated with 1 ml of lysis buffer containing 50 mM Tris-HCl, 150 mM NaCl, 5 mM EDTA, 1% Lubrol, and a protease inhibitor mixture for 20 min on ice. The insoluble fraction was discarded after centrifugation at 10,000×*g* for 20 min at 4 °C. After centrifugation, the lysates were incubated with different antibodies and Sepharose beads conjugated with protein A/G for 10 h at 4 °C. The Sepharose beads were washed 4 times with the lysis buffer, and the proteins were eluted from the beads in sample buffer at 95 °C for 5 min. Subsequently, the proteins were detected by an immunoblotting (IB) analysis.

### Peptides

All peptides used in this study were synthesized by Bankpeptide Biological Technology Company (Hefei, China). The peptides were HPLC-purified to reach a purity of more than 95%. All peptides were stored in a powder form and freshly diluted to 20 μM or 10 mg/ml for use in the cell- or animal-based experiments.

### Cell Viability Assessment

Cell death was quantitatively assessed using the LDH-Cytotoxicity Colorimetric Assay Kit (BioVision, USA), and the proportion of necrotic cells was detected (propidium iodide and Hoechst 33342 staining). LDH release was defined based on the ratio of LDH in the media to the total LDH and was normalized to the control. The quantification of the necrotic/healthy cells was performed by costaining the samples with 5 μM Hoechst 33342 and 2 μM propidium iodide, followed by blinded counts. The percentage of cell death was determined as the ratio of the number of neurons stained with propidium iodide to the number stained with Hoechst 33342.

### Statistical Analysis

The data are reported as the means ± SD or means ± SEM, and the analysis was performed using GraphPad Prism 6.0 software. The significance of the differences was determined by Student’s *t* test, one-way analysis of variance (ANOVA) followed by Tukey-Kramer’s post hoc test, repeated-measures one-way ANOVA, the Wilcoxon rank sum test, or the Kruskal-Wallis test unless otherwise specified. Statistical significance was defined as *p <* 0.05.

## Results

### NDRG2 Knockout Exacerbates Cerebral Ischemic Damage and Glutamate Excitotoxicity

**Fig. 1:**
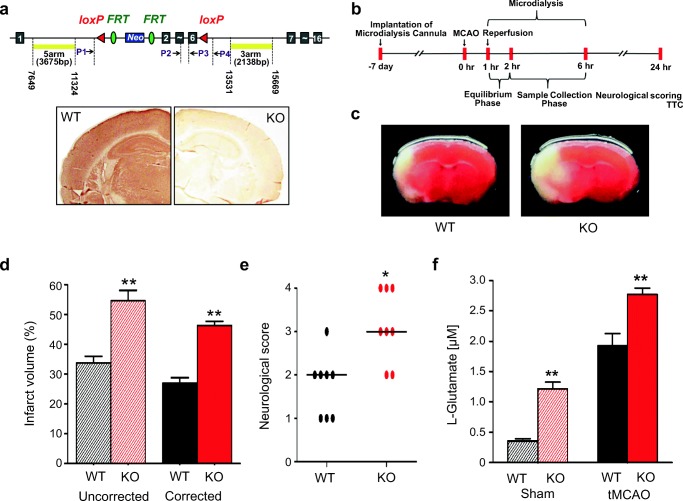
NDRG2 deficiency exacerbates focal cerebral ischemia and promotes interstitial glutamate accumulation. **a** Schematic representation of the generation of the *Ndrg2*^*−/−*^ (KO) mice and immunohistochemical identification using an NDRG2-specific antibody in WT and KO mouse brains. **b** The flow diagram of tMCAO, microdialysis, behavioral and infarct volume analysis. **c**–**f** WT and KO mice were subjected to MCAO and 24 h of reperfusion (*n* = 8). Representative coronal brain sections stained with TTC (**c**), quantification of the infarct volume (**d**), and neurological scoring (**e**) after tMCAO. (**f**) Microdialysis samples were collected from extracellular fluid in the striatum after tMCAO as indicated. The glutamate concentrations in the microdialysis samples were measured by HPLC. *p < 0.05, **p < 0.01, Wilcoxon rank sum test (**d**, **e**). **p < 0.01, Student’s *t* test (**f**). Error bars, mean ± SD (**d**, **f**); Horizontal bars, medians (**e**)

NDRG2 was reported as an early-stage stress-responsive gene, and its expression can be induced by several types of stimulation, including cerebral ischemia [22]. Here, we also found that the expression of NDRG2 increased after cerebral ischemia in vivo or glutamate stimulation in vitro (Fig. S2). To explore the potential role of NDRG2 in cerebral ischemia, mice carrying an *NDRG2* deletion that targeted exons 2–6 (Fig. 1a, top panel) were generated. These *Ndrg2*^*−/−*^ mice were fully fertile and grew at a normal rate; no detectable brain structural differences were observed between the 8-week-old *Ndrg2*^*−/−*^ mice and their WT littermates (Fig. 1a, bottom panel). We first examined whether NDRG2 is associated with the changed neurological outcomes in ischemia following a timeline (Fig. 1b). The infarct volume after MCAO and 24-h reperfusion was markedly larger in the *Ndrg2*^*−/−*^ brains than that in the WT brains (Fig. 1c, d). We also observed that the *Ndrg2*^*−/−*^ mice exhibited significantly increased neurological deficits after ischemia and 24-h reperfusion (Fig. 1e). Consistent with the 24-h reperfusion data, the KO mice exhibited an increased infarct volume compared with WT mice after 3 and 7 days of reperfusion (Fig. S3a and b). In addition, although the neurological outcome 7 days after reperfusion was better than that at 24 h or 3 days, the KO mice exhibited increased neurological deficits compared with the WT mice (Fig. S3c and d). No lesions were found in the *Ndrg2*^*−/−*^ mice or WT mice after the sham operations (Fig. S3e).

The sudden cessation of cerebral blood flow is accompanied by a marked increase in glutamate release [28]. Glutamate excitotoxicity plays a prominent role in ischemic cerebral injury [29]. We previously found increased glutamate in the brains of *Ndrg2*^*−/−*^ mice, which exhibit attention deficit/hyperactivity-like behavior [25]. Therefore, we examined the interstitial glutamate levels in the *Ndrg2*^*−/−*^ mice brains after ischemia by microdialysis. The levels of interstitial glutamate were significantly higher in the striatum of the *Ndrg2*^*−/−*^ mice than those in the striatum of the WT mice both at the baseline level and after tMCAO treatment (Fig. 1f). These data suggest that NDRG2 plays a neuroprotective role in cerebral ischemia by regulating glutamate transport.

### Impaired Glutamate Uptake in Ndrg2^−/−^ Astrocytes

**Fig. 2:**
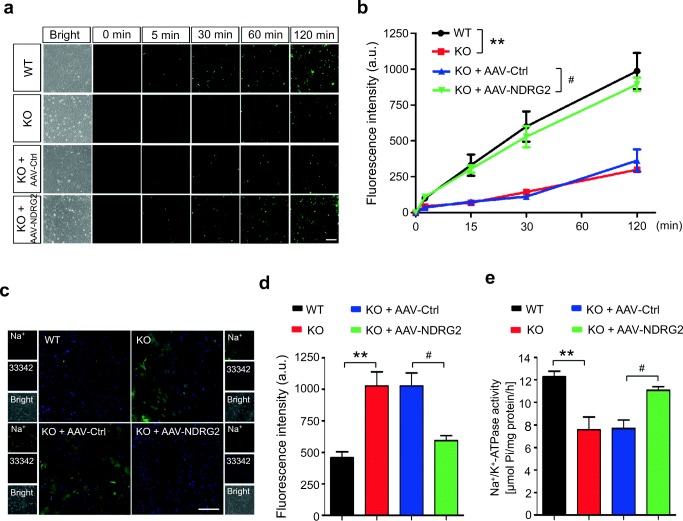
NDRG2 is required for the sodium-dependent glutamate uptake into astrocytes. WT astrocytes, KO astrocytes, and KO astrocytes infected with a control virus (AAV-Ctrl) or the NDRG2 virus (AAV-NDRG2). **a** Representative photomicrographs of FITC-coupled glutamate uptake at various time points. Scale bar = 100 μm. **b** Green fluorescence intensity of FITC-coupled glutamate in each group shown in a. Data are presented as the means ± SEM of three independent experiments, each of which was performed in 5 fields of view and evaluated using repeated-measures one-way ANOVA. **p < 0.01 versus WT, ^#^*p* < 0.05 versus KO + AAV-Ctrl. **c** Representative fluorescence images showing CoroNa Green (sodium indicator) trapped inside astroglial cytoplasm and counterstained with Hoechst 33342. Scale bar = 100 μm. **d** Green fluorescence intensity of CoroNa in each group shown in **d**. Data are presented as the means ± SEM of three independent experiments, each performed in 5 fields of view and evaluated using one-way ANOVA followed by Tukey-Kramer’s post hoc test. **p < 0.01 versus WT, ^#^*p* < 0.05 versus KO + AAV-Ctrl. **e** Na^+^/K^+^-ATPase activity was determined following the experimental procedure in different astrocytes. Data are expressed as the means ± SEM from three independent determinations, each of which was performed in quadruplicate and evaluated using one-way ANOVA followed by Tukey-Kramer’s post hoc test. **p < 0.01 versus WT, ^#^*p* < 0.05 versus KO + AAV-Ctrl

Because NDRG2 is mainly expressed in astrocytes [17–19], we investigated the glutamate uptake ability in cultured *Ndrg2*^*−/−*^ astrocytes. FITC-coupled glutamate was used to visualize dynamic glutamate trafficking. The speed and amount of the FITC-coupled glutamate uptake were significantly lower in the *Ndrg2*^*−/−*^ astrocytes than those in the WT astrocytes at various time points (Fig. 2a, b). We constructed an adeno-associated virus (AAV) that specifically expresses NDRG2 in astrocytes. Strikingly, the NDRG2 rescue treatment reversed the decreased FITC-coupled glutamate uptake into *Ndrg2*^*−/−*^ astrocytes (Fig. 2a, b). Here, we observed that NDRG2 levels are related to glutamate uptake in real time, although we previously also found decreased glutamate clearance in cultured *Ndrg2*^*−/−*^ astrocytes by indirectly detecting the remnant glutamate in the culture medium [25].

Sodium-dependent and sodium-independent glutamate uptake in astrocytes are important for preventing excitotoxicity [6]. We previously found that NDRG2 can interact with Na^+^/K^+^-ATPase β1 [30], which is required for Na^+^ transport. To determine whether the NDRG2-related glutamate uptake is sodium-dependent, we examined the intracellular Na^+^ concentration and the activity of Na^+^/K^+^-ATPase in cultured astrocytes. The Na^+^ fluorescence imaging revealed a substantially higher amount of intracellular Na^+^ in the *Ndrg2*^*−/−*^ astrocytes than in the WT astrocytes (Fig. 2c, d), which is suggestive of a decreased transmembrane Na^+^ gradient in the *Ndrg2*^*−/−*^ astrocytes. The *NDRG2*-silenced astrocytes showed a reduction in the activity of Na^+^/K^+^-ATPase (Fig. 2e), which is consistent with our previous study in salivary epithelial cells [30]. In addition, attenuated intracellular Na^+^ and increased Na^+^/K^+^-ATPase activity were detected following the rescue of NDRG2 expression via the AAV treatment in the *Ndrg2*^*−/−*^ astrocytes. Together, these results indicate that NDRG2 is required for the maintenance of sodium-dependent astroglial glutamate uptake.

Because stroke results from a combination of excitotoxicity and energy pump failure, we next tested astrocytic Na^+^/K^+^-ATPase activity and glutamate uptake under oxygen-glucose deprivation/reoxygenation (OGD/OGR) conditions. Na^+^/K^+^-ATPase activity and glutamate uptake were significantly reduced in both KO and WT astrocytes after 2 h of OGD and 6 h of OGR. Moreover, KO astrocytes exhibited a more severe impairment in Na^+^/K^+^-ATPase activity and glutamate uptake upon OGD compared with WT astrocytes (Fig. S4a and b). Therefore, these data suggest that NDRG2 deficiency aggravates the disruption of Na^+^/K^+^-ATPase function and Na^+^/K^+^-ATPase-associated glutamate uptake under energy blocking.

### Na^+^/K^+^-ATPase β1 Is Involved in NDRG2-Regulated Glutamate Uptake

**Fig. 3:**
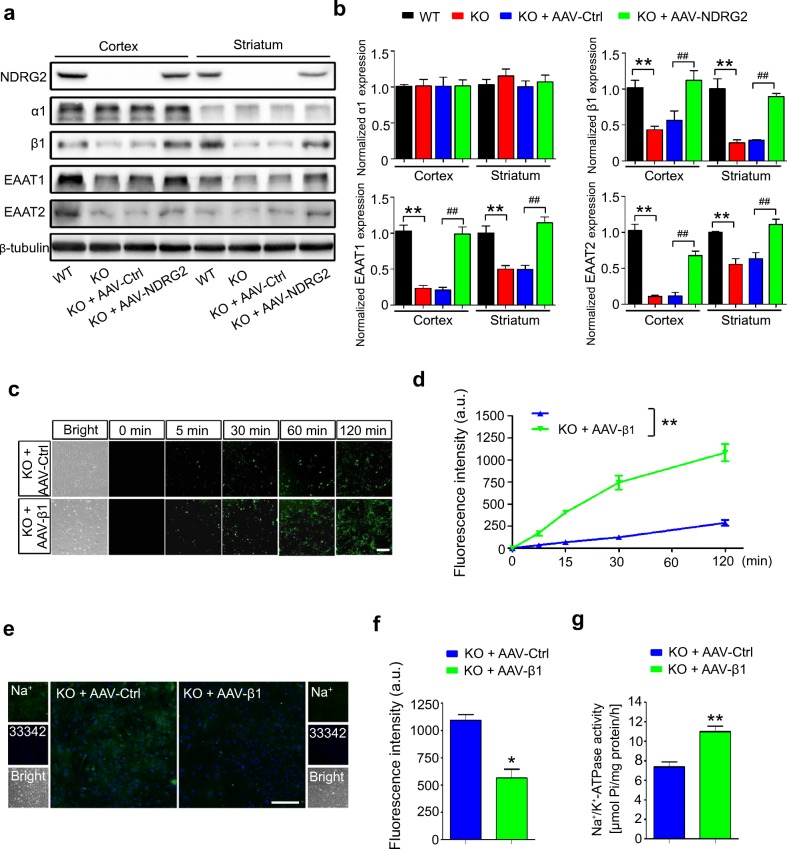
NDRG2 promotes glutamate uptake by regulating Na^+^/K^+^-ATPase β1. **a** Representative immunoblots of NDRG2, Na^+^/K^+^-ATPase α1 (α1), Na^+^/K^+^-ATPase β1 (β1), EAAT1, and EAAT2 proteins from the cortex and striatum of WT, KO, and KO mice injected with a control virus (AAV-Ctrl) or the *NDRG2* virus (AAV-NDRG2). **b** The data shown in **a** were quantified and normalized to β-tubulin. Data are presented as the means ± SEM of three independent experiments and were evaluated using one-way ANOVA followed by Tukey-Kramer’s post hoc test. **p < 0.01 versus WT, ^##^*p* < 0.01 versus KO + AAV-Ctrl. **c** Representative photomicrographs of FITC-coupled glutamate uptake in KO astrocytes infected with a control virus (AAV-Ctrl) or the Na^+^/K^+^-ATPase β1 virus (AAV-β1) at various time points. Scale bar = 100 μm. **d** Green fluorescence intensity of FITC-coupled glutamate in each group shown in **c**. Data are presented as the means ± SEM of three independent experiments, each of which was performed in 5 fields of view and evaluated using repeated-measures one-way ANOVA. **p < 0.01 versus KO + AAV-Ctrl. **e** Representative fluorescence images showing CoroNa Green (sodium indicator) trapped inside the KO astrocytes infected with AAV-Ctrl or AAV-β1 and counterstained with Hoechst 33342. Scale bar = 100 μm. **f** Green fluorescence intensity of CoroNa in each group shown in **e**. Data are presented as the means ± SEM of three independent experiments, each of which was performed in 5 fields of view and evaluated using Student’s *t* test. *p < 0.05 versus KO + AAV-Ctrl. **g** Na^+^/K^+^-ATPase activity was determined following the experimental procedure in different astrocytes. Data are expressed as the means ± SEM of three independent determinations, each of which was performed in quadruplicate and evaluated using Student’s *t* test. **p < 0.01 versus KO + AAV-Ctrl

To determine the mechanism by which an NDRG2 deficiency leads to impaired glutamate uptake, we examined the expression levels of the molecules implicated in this process. The IB analysis revealed that the levels of EAAT1, EAAT2, and Na^+^/K^+^-ATPase β1, but not the level of Na^+^/K^+^-ATPase α1, were markedly decreased in the cortex and striatum of the *Ndrg2*^*−/−*^ mice (Fig. 3a, b). These results suggest that NDRG2 stabilizes the Na^+^/K^+^-ATPase β1 protein in astrocytes, consistent with our previous report indicating that NDRG2 inhibited the ubiquitination and degradation of Na^+^/K^+^-ATPase β1 in salivary epithelial cells [30]. Next, we injected the NDRG2 AAV into *Ndrg2*^*−/−*^ mice via the lateral ventricles, which specifically rescued the astrocytic NDRG2 expression in the brain, including striatal ischemic penumbra (Fig. S5). The expression levels of Na^+^/K^+^-ATPase β1, EAAT1, and EAAT2 were remarkably recovered by restoring the expression of NDRG2 in the cortex and striatum of the *Ndrg2*^*−/−*^ mice (Fig. 3a, b).

We previously screened 12 potential interaction partners of NDRG2 using the yeast two-hybrid system [30]; of these candidate partners, three were identified as Na^+^/K^+^-ATPase β1 (Table S2). We hypothesized that Na^+^/K^+^-ATPase β1 is involved in NDRG2-mediated glutamate uptake because Na^+^/K^+^-ATPase β1 plays a critical role in the membrane translocation of Na^+^/K^+^-ATPase and is required for enzymatic activity [31, 32]. We confirmed that exogenous NDRG2 interacted with exogenous Na^+^/K^+^-ATPase β1 in HEK293 cells (Fig. S6a). We further detected endogenous NDRG2 coprecipitated with endogenous Na^+^/K^+^-ATPase β1 in cultured astrocytes (Fig. S6b).

In addition, we constructed an AAV that specifically expresses Na^+^/K^+^-ATPase β1 in astrocytes. The decreased uptake of FITC-coupled glutamate into *Ndrg2*^*−/−*^ astrocytes was significantly rescued by the Na^+^/K^+^-ATPase β1 AAV treatment (Fig. 3c, d). Attenuated intracellular Na^+^ and increased activity of Na^+^/K^+^-ATPase were detected following the rescue of NDRG2 expression in the *Ndrg2*^*−/−*^ astrocytes (Fig. 3e–g). Together, these results suggest that Na^+^/K^+^-ATPase β1 is critical for the expression and function of EAAT1/2 and underlies the NDRG2-regulated glutamate uptake.

### A Na^+^/K^+^-ATPase β1 Peptide Attenuates NDRG2-Mediated Glutamate Uptake

**Fig. 4:**
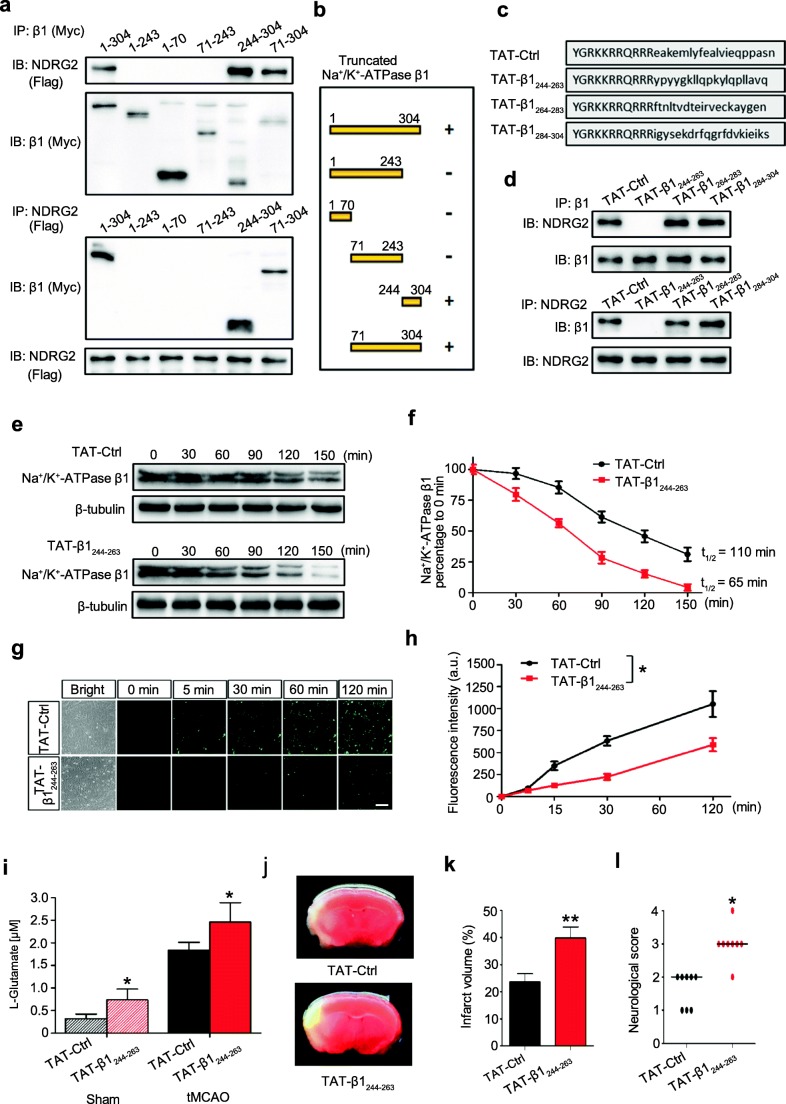
Dissociation of the NDRG2-Na^+^/K^+^-ATPase β1 interaction abolishes the NDRG2-mediated cerebral protection. **a** Co-IP analysis of NDRG2 and Na^+^/K^+^-ATPase β1 (β1). Flag-tagged full-length NDRG2 was cotransfected with Myc-tagged full-length Na^+^/K^+^-ATPase β1 or its truncated mutants into HEK293 cells for 48 h. IP was performed using an anti-Myc antibody. Precipitated proteins were analyzed by IB with an anti-Flag antibody or anti-Myc antibody (upper panel). IP was also performed using an anti-Flag antibody and analyzed by IB with anti-Myc and anti-Flag antibodies (lower panel). Similar results were observed in three independent experiments. **b** Schematic of the interactions between Myc-tagged full-length Na^+^/K^+^-ATPase β1 and its different truncation mutants with Flag-tagged full-length NDRG2. **c** Synthetic peptides TAT-β1_244–263_, TAT-β1_264–283_, and TAT-β1_284–304_ and the control peptide TAT-Ctrl with a scrambled sequence. **d** Co-IP analysis of endogenous NDRG2 and Na^+^/K^+^-ATPase β1 was performed following different peptide treatments in cultured astrocytes for 2 h. IP and IB analyses were performed as indicated. Similar results were obtained from three independent experiments. **e** Representative immunoblots of Na^+^/K^+^-ATPase β1 following TAT-Ctrl or TAT-β1_244–263_ administration with 100 μM emetine treatment. β-tubulin served as a loading control. **f** Relative levels of Na^+^/K^+^-ATPase β1 to β-tubulin shown in **e** were quantified by densitometry, and the percentage of the value at 0 min was calculated at various time points (from 0 to 150 min). Data are shown as the means ± SEM of three independent experiments. **g** Representative photomicrographs of FITC-coupled glutamate uptake after the TAT-Ctrl or TAT-β1_244–263_ treatment in cultured astrocytes. Scale bar = 100 μm. Similar results were obtained in each of three independent experiments. **h** Green fluorescence intensity of FITC-coupled glutamate in each group shown in **g**. Data are presented as the means ± SEM of three independent experiments, each of which was performed in 5 fields of view and evaluated using repeated-measures one-way ANOVA. *p < 0.05 versus TAT-Ctrl. **i**–**l** Mice were intravenously injected with TAT-Ctrl or TAT-β1_244–263_ 2 h before tMCAO (*n* = 8). **i** The concentrations of glutamate in the microdialysis samples were measured using HPLC. Student’s *t* test. *p < 0.05 versus TAT-Ctrl. Representative coronal brain sections stained with TTC (**j**), quantification of infarct volume (**k**), and neurological score (**l**) after tMCAO. **i**, **k**, Student’s *t* test; **l**, Wilcoxon test. *p < 0.05, **p < 0.01 versus TAT-Ctrl. Error bars, mean ± SD (**i**, **k**); horizontal bars, medians (**l**)

To identify the key segment of Na^+^/K^+^-ATPase β1 binding with NDRG2, we truncated Na^+^/K^+^-ATPase β1 into peptides of different lengths. Deletion mapping using these truncated peptides of Na^+^/K^+^-ATPase β1 to conduct the coimmunoprecipitation showed that the region encompassing amino acid residues 244–304 is critical for the interaction between Na^+^/K^+^-ATPase β1 and NDRG2 (Fig. 4a, b). However, a peptide containing 60 amino acids is still too large. Therefore, the 60 amino acid residues were subdivided into three peptides, each containing 20 amino acid residues. We further combined each peptide with an HIV trans-activator of transcription (TAT: YGRKKRRQRRR) sequence, which allows the peptide to cross the blood-brain barrier and cell membrane. Therefore, we synthesized three peptides containing a TAT sequence, which allows the peptide to cross the blood-brain barrier and cell membrane [33, 34], and subdivided regions of the 244–304 peptide sequence (Fig. 4c). These peptides were individually added to cultured WT astrocytes. TAT-β1_244–263_, but not TAT-β1_264–283_ or TAT-β1_284–304_, competitively blocked the binding of NDRG2 to endogenous Na^+^/K^+^-ATPase β1 in astrocytes (Fig. 4d).

Therefore, we hypothesize that the TAT-β1_244–263_ peptide can competitively combine with endogenous NDRG2 and disturb the binding of NDRG2-Na^+^/K^+^-ATPase β1, leading to faster degradation of endogenous Na^+^/K^+^-ATPase β1. Next, we examined the protein stability of Na^+^/K^+^-ATPase β1 and glutamate uptake after TAT-β1_244–263_ treatment in the astrocytes. The half-life of endogenous Na^+^/K^+^-ATPase β1 following the TAT-β1_244–263_ treatment (65 min) was much shorter than that following the TAT-Ctrl treatment (110 min) (Fig. 4e, f), indicating that endogenous Na^+^/K^+^-ATPase β1 is less stable when unable to interact with NDRG2. In addition, the speed and amount of the astroglial glutamate uptake were significantly reduced after the TAT-β1_244–263_ administration (Fig. 4g, h). Increased intracellular Na^+^ and reduced Na^+^/K^+^-ATPase activity were also detected in the cultured astrocytes after the TAT-β1_244–263_ treatment (Fig. S7). Altogether, these findings suggest that the NDRG2-Na^+^/K^+^-ATPase β1 interaction is required not only for the stability of Na^+^/K^+^-ATPase β1 but also for sodium-dependent glutamate uptake into astrocytes.

We further investigated whether the NDRG2-Na^+^/K^+^-ATPase β1 interaction is required for the NDRG2-mediated neuronal protection against glutamate excitotoxicity in vitro. The astrocytes were pretreated with TAT-β1_244–263_ or TAT-Ctrl for 2 h and then indirectly cocultured with neurons. The glutamate clearance was markedly decreased in the TAT-β1_244–263_ pretreated group compared with that in the TAT-Ctrl pretreated group after 2 h of glutamate exposure (Fig. S8a). We also observed significant increases in the LDH release and neuronal death in the TAT-β1_244–263_ pretreated group after glutamate administration (Fig. S8b and c).

To determine whether the NDRG2-Na^+^/K^+^-ATPase β1 association is required for the NDRG2-mediated neuroprotection in vivo, we performed tMCAO experiments. The mice were intravenously injected with either TAT-Ctrl or TAT-β1_244–263_ 2 h before tMCAO (Fig. S9a). Consistent with the results of the in vitro experiments, TAT-β1_244–263_ triggered a dissociation of the NDRG2-Na^+^/K^+^-ATPase β1 interaction in the cortex and striatum of the mouse brain (Fig. S9b). Compared with TAT-Ctrl, TAT-β1_244–263_ induced a significant increase in interstitial glutamate after ischemia (Fig. 4i). The brain infarct volume was notably larger in the TAT-β1_244–263_-injected mice compared with the TAT-Ctrl-injected mice after tMCAO (Fig. 4j, k). Consistently, we observed that the ischemia-associated neurological deficits were significantly worsened in the TAT-β1_244–263_-injected mice compared with those in the TAT-Ctrl-injected mice (Fig. 4l). Altogether, these results indicate that the NDRG2-Na^+^/K^+^-ATPase β1 interaction is functionally necessary for NDRG2-mediated neuroprotection against glutamate excitotoxicity.

### Astroglial NDRG2 Is Required for Neuronal Survival under Glutamate Excitotoxicity

**Fig. 5:**
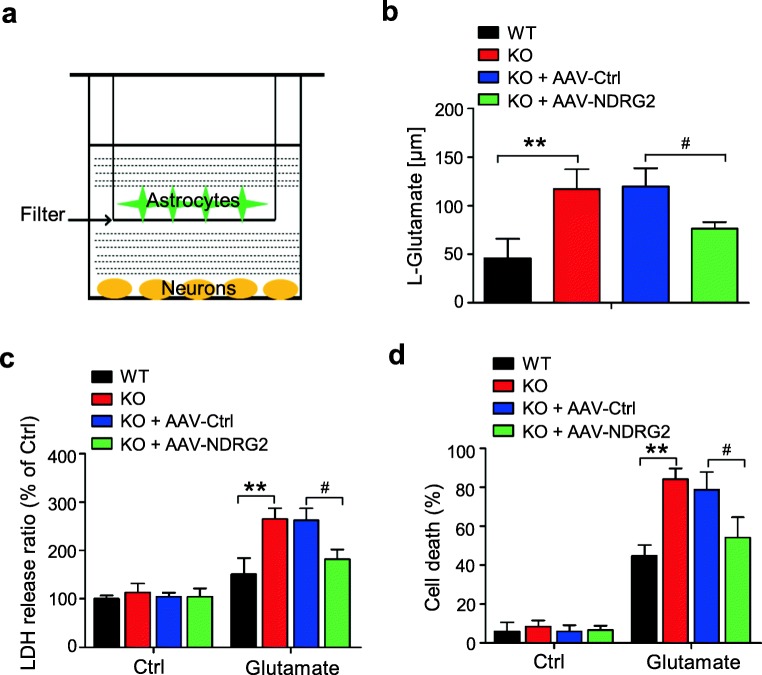
NDRG2 deletion increases neuronal death following a glutamate challenge in vitro. Neurons from WT mice were cocultured with WT astrocytes, KO astrocytes, and KO astrocytes infected with a control virus (AAV-Ctrl) or the NDRG2 virus (AAV-NDRG2). **a** Schematic diagram of the neuron-astrocyte indirect cocultures. Astrocytes were seeded on the top filter, and WT neurons were planted in the bottom chamber. **b** Cocultures were treated with 200 μM glutamate, and the glutamate levels in the culture medium were measured at various time points. Data represent the means ± SEM of three independent determinations, each of which was performed in quadruplicate and evaluated using one-way ANOVA followed by Tukey-Kramer’s post hoc test. **p < 0.01 versus WT, ^#^*p* < 0.05 versus KO + AAV-Ctrl. Quantification of LDH release from neurons (**c**) and neuronal death percentage (**d**) after 2 h of glutamate treatment and another 24 h of further culture without glutamate. Data represent the means ± SEM of three independent determinations, each of which was performed in quadruplicate and evaluated using one-way ANOVA followed by Tukey-Kramer’s post hoc test. **p < 0.01 versus WT, ^#^*p* < 0.05 versus KO + AAV-Ctrl

Next, we used mixed neuron-astrocyte cultures to detect whether astroglial NDRG2 is associated with neuronal survival following a glutamate challenge in vitro. The mixed cultures were obtained from indirect neuron and astrocyte cocultures (WT or *Ndrg2*^*−/−*^ astrocytes were plated in the upper chamber, and WT neurons were seeded in the lower chamber), allowing the two cell types to share the same diffusible materials but remain divided by a physical filter (Fig. 5a). These indirect neuron-astrocyte cocultures were treated with 200 μM glutamate for 2 h. The remaining glutamate in the medium of the *Ndrg2*^*−/−*^ astrocyte-neuron cocultures was more than two times higher than that in the medium of the WT astrocyte-neuron cocultures (Fig. 5b). This finding is consistent with the FITC-coupled glutamate uptake results in monocultures of *Ndrg2*^*−/*^ astrocytes (Fig. 2a, b).

We also examined the cell viability of the neurons. The *Ndrg2*^*−/−*^ astrocyte-neuron cocultures showed higher lactate dehydrogenase (LDH) release from neurons (Fig. 5c) and more neuronal death (Fig. 5d) than the WT astrocyte-neuron cocultures after glutamate treatment. The increased NDRG2 expression in the *Ndrg2*^*−/−*^ astrocytes effectively rescued the glutamate clearance (Fig. 5b) and attenuated the neuronal LDH release and neuronal death (Fig. 5c, d). These results indicate that NDRG2 protects neurons from glutamate excitotoxicity by promoting astroglial glutamate clearance.

### NDRG2 Confers Neuroprotection Against Ischemia

**Fig. 6:**
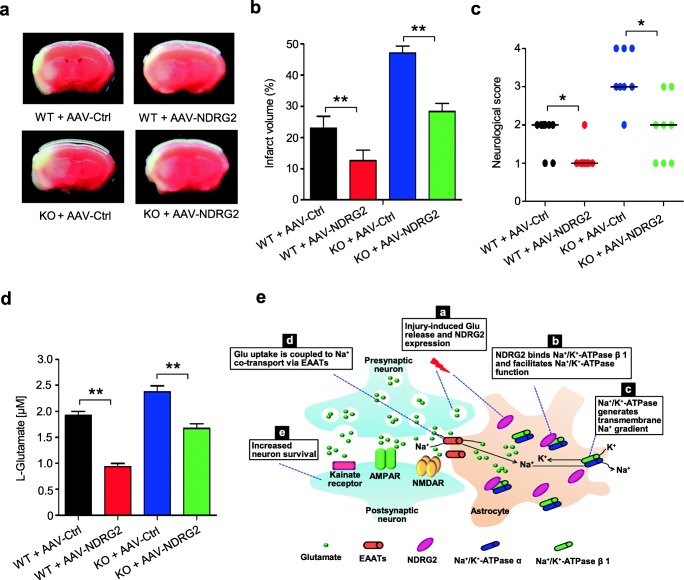
NDRG2 protects the brain from ischemic stroke. WT and KO mice injected with a control virus (AAV-Ctrl) or the *NDRG2* virus (AAV-NDRG2) were subjected to tMCAO. Representative coronal brain sections stained with TTC (**a**), quantification of infarct volume (**b**), and neurological score (**c**) after tMCAO (*n* = 8). **b** One-way ANOVA followed by Tukey-Kramer’s post hoc test; **c** Kruskal-Wallis test. **p < 0.05, **p < 0.01 versus WT + AAV-Ctrl or KO + AAV-Ctrl. **d** Microdialysis samples were collected from striatal extracellular fluid after tMCAO as indicated. The concentrations of glutamate in the microdialysis samples were measured by HPLC. Data were evaluated using one-way ANOVA followed by Tukey-Kramer’s post hoc test. **p < 0.01 versus WT + AAV-Ctrl or KO + AAV-Ctrl. Error bars, mean ± SD (**b**, **d**); horizontal bars, medians (**c**). **e** A working model of astroglial NDRG2-mediated neuroprotection following brain ischemia

Glutamate-induced excitotoxicity plays a prominent role in various neurological disorders, such as ischemic cerebral injury [29]. We therefore explored whether an NDRG2 treatment could improve neurological function after brain ischemia. An intracerebroventricular injection of the NDRG2 AAV effectively decreased the infarct volume and neurological deficits in both WT and *Ndrg2*^*−/−*^ mice after tMCAO (Fig. 6a–c).

Thus, we investigated the interstitial glutamate levels in the mouse brain after ischemia. The increase in interstitial glutamate was significantly attenuated after injection of the NDRG2 AAV into WT and *Ndrg2*^*−/−*^ brains after tMCAO (Fig. 6d). In addition, the NDRG2 AAV injection effectively decreased the interstitial glutamate levels in the *Ndrg2*^*−/−*^ brains after the sham operation (Fig. S10).

Collectively, these results indicate that astroglial NDRG2 plays a neuroprotective role in cerebral ischemia and is a potential therapeutic target in brain stroke.

## Discussion

The results of the present study revealed that NDRG2 plays an important role in maintaining the synaptic glutamate balance and alleviating glutamate excitotoxicity. NDRG2 is required for astroglial glutamate uptake from cerebral interstitial fluid, as it interacts with Na^+^/K^+^-ATPase β1, which protects the brain from ischemic stroke.

In mammalian brains, NDRG2 is primarily expressed in astrocytes and has been considered a new marker of mature astrocytes [17–19]. However, the functions of NDRG2 in astrocytes remain largely unknown. Astrocytes are the predominant cells contributing to the removal of excitatory glutamate from the synaptic space [35]. Here, we found that *Ndrg2*^*−/−*^ mice exhibited increased interstitial glutamate in the brain and that impaired astroglial glutamate uptake underlies the glutamate accumulation. Many studies have focused on the modulation of glutamate uptake by astrocytic EAATs, which are driven by the transmembrane Na^+^ gradient [11, 36, 37]. In *Xenopus laevis* oocytes, NDRG2 stimulates an amiloride-sensitive Na^+^ current by regulating the epithelial sodium channel [38]. We determined that NDRG2 is required for the Na^+^/K^+^-ATPase-mediated Na^+^ current in salivary cells [39]. Here, we demonstrated that *NDRG2* silencing resulted in a build-up of intracellular Na^+^ and a decrease in Na^+^/K^+^-ATPase activity in astrocytes. Therefore, we speculate that NDRG2 regulates the function of EAATs through Na^+^/K^+^-ATPase. Although most mammalian cells maintain a very stable intracellular Na^+^ concentration under physiological conditions, in astrocytes, the Na^+^ concentrations widely fluctuate to buffer neighboring extracellular Na^+^ derived from neurons and other cells in the brain [40–42], which may explain why the cultured *Ndrg2*^*−/−*^ astrocytes could survive a high intracellular Na^+^ concentration.

We previously found that NDRG2 interacts with and stabilizes Na^+^/K^+^-ATPase β1 [31, 43]. In the present study, we developed a Na^+^/K^+^-ATPase β1 peptide that could dissociate the NDRG2- Na^+^/K^+^-ATPase β1 interaction, sequentially leading to a disruption in sodium-dependent glutamate uptake and glutamate excitotoxicity. Na^+^/K^+^-ATPase β1 plays important roles in the membrane translocation and enzyme function of the alpha subunit of Na^+^/K^+^-ATPase [31, 43]. The Na^+^/K^+^-ATPase α subunit and EAATs are physically associated within a macromolecular complex in the plasma membrane [15]. Thus, a dynamic NDRG2-Na^+^/K^+^-ATPase β1-EAAT complex may modulate sodium-dependent glutamate uptake in astrocytes [25].

The NDRG2-mediated glutamate uptake and glutamate homeostasis are not only required for the physiological function of the brain but also enhance the tolerance of the brain to glutamate excitotoxicity under neuropathological conditions. While NDRG2 is normally expressed at a moderate level in the brain, its expression was notably increased under excitotoxic conditions, including cerebral ischemia and trauma [22, 24, 44]. The upregulated NDRG2 expression may reflect the brain’s attempt to maintain the glutamate balance and protect itself from excitotoxicity under restricted blood supply and other neuropathological conditions. Increasing or rescuing the expression of NDRG2 in WT and *Ndrg2*^*−/−*^ mice significantly reduces the infarct volume, neurological outcome, and interstitial glutamate levels, suggesting a potentially therapeutic target for brain ischemia. A recent study reported that the loss of NDRG2 increases astrocytic MMP activity and BBB permeability, which exacerbate subsequent brain damage after permanent focal cerebral ischemia [45]. This is consistent with the function of NDRG2 as a neuroprotective protein observed in the present study, although we focused on astrocyte-mediated glutamate uptake but not astrocyte-associated blood cell infiltration and BBB permeability.

Here, we used the *Ndrg2*^*−/−*^ mice to explore the underlying function of NDRG2 in cerebral ischemia and concluded that NDRG2 plays a neuroprotective role. However, the results of another report on the role of NDRG2 in stroke [46] were contradictory to our conclusion. In that study, the OGD model in vitro was used to mimic the situation in which astrocytes suffered from ischemic-reperfusion injury, and NDRG2 silencing significantly reduced astrocytic reactive oxygen species production and apoptosis after exposure to OGR. This discrepancy could be attributed to the difference between in vivo and in vitro pathological models. Thus, more studies are needed to clarify this issue in the future.

In addition, in a mouse model of cortical stab injury, the NDRG2 expression was elevated in astrocytes surrounding the wounded area and deletion of NDRG2 resulted in a lower induction of reactive astrogliosis and inflammatory response in the injured cortex [44]. NDRG2 also participated in other signaling pathways relating to neuronal death. In a previous study [47], the authors used phospho-peptide library screening and found that phosphorylation of NDRG2 on Ser350 by DAPK1 could be a novel mechanism of NDRG2 activation and could be involved in neuronal cell death. This result indicates that NDRG2 may play multiple roles in the central nervous system.

In summary, our results support a scenario (Fig. 6e) of NDRG2-mediated neuroprotection. In response to cerebral ischemia, excessive glutamate is released from the presynaptic membrane, and astroglial NDRG2 is rapidly upregulated. Increased NDRG2 interacts with Na^+^/K^+^-ATPase β1 and facilitates the Na^+^/K^+^-ATPase-triggered transmembrane Na^+^ gradient, leading to the cotransportation of both glutamate and Na^+^ into astrocytes via the EAATs. Subsequently, glutamate receptors are appropriately activated, but not overloaded, to maintain excitatory synaptic transmission. Therefore, the accelerated glutamate clearance protects the neurons from further glutamate excitotoxicity. Our findings provide a potential target for future interventions for glutamate excitotoxicity-associated diseases such as ischemia.

## Electronic Supplementary Material

ESM 1 (DOCX 4136 kb)
