# Supplementary material for: NDRG2 Protects the Brain from Excitotoxicity by Facilitating Interstitial Glutamate Uptake
Source: Transl Stroke Res. 2019 Jun 27;11(2):214–27. doi: 10.1007/s12975-019-00708-9 (PMC7067740; doi:10.1007/s12975-019-00708-9)
Supplement: Supplementary file 1 — (DOCX 4136 kb) [file 12975_2019_708_MOESM1_ESM.docx]

**NDRG2 protects the brain from** **excitotoxicity by facilitating interstitial** **glutamate uptake**

Anqi Yin, Hang Guo, Liang Tao, Guohong Cai, Yazhou Wang, Libo Yao, Lize Xiong, Jian Zhang, and Yan Li

**Supplementary materials index**

1. Supplementary Figures 1-10

2. Supplementary Tables 1-2

**Supplementary figures**


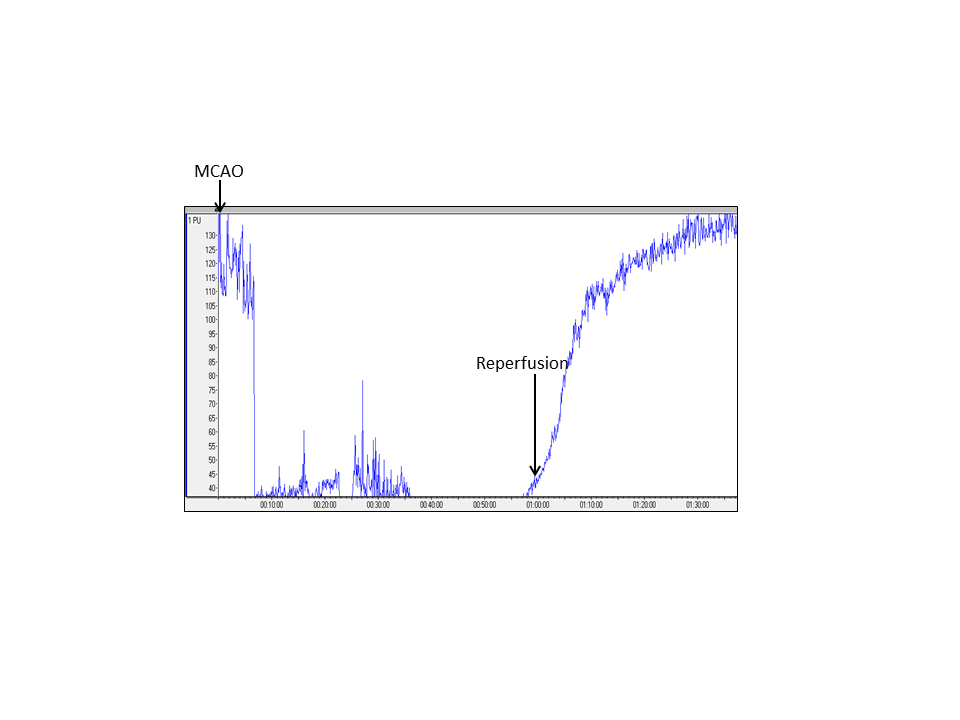


**Fig. S1** Representative trace of cerebral blood flow in mice subjected to 60 minutes of MCAO, followed by reperfusion.


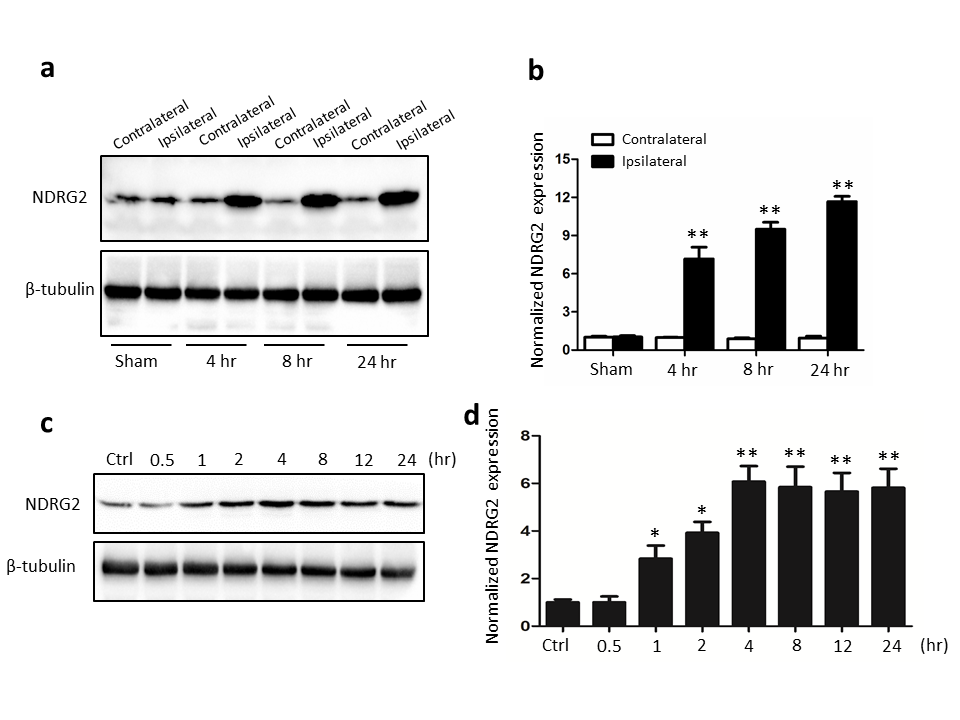


**Fig. S2** Analysis of the expression of NDRG2 after cerebral ischemia *in vivo* and glutamate stimulation *in vitro*. (a) Representative immunoblots of NDRG2 protein after the mice were subjected to 60 minutes of MCAO and 4, 8 and 24 hours of reperfusion. (b) Data shown in A were normalized to β-tubulin and quantified by optical density. Data represent the means ± SEM of three independent experiments and were analyzed using one-way ANOVA followed by Dunnett’s post hoc test. ^**^*p* < 0.01 versus Sham ipsilateral group. (c) Representative immunoblots of NDRG2 in cultured astrocytes treated with 200 μM glutamate at various time points. (d) Data shown in C were normalized to β-tubulin and quantified by optical density. Ctrl, control; hr, hour. Data represent the mean ± SEM of three independent experiments and were analyzed using one-way ANOVA followed by Dunnett’s post hoc test. ^*^*p* < 0.05, ^**^*p* < 0.01 versus Ctrl


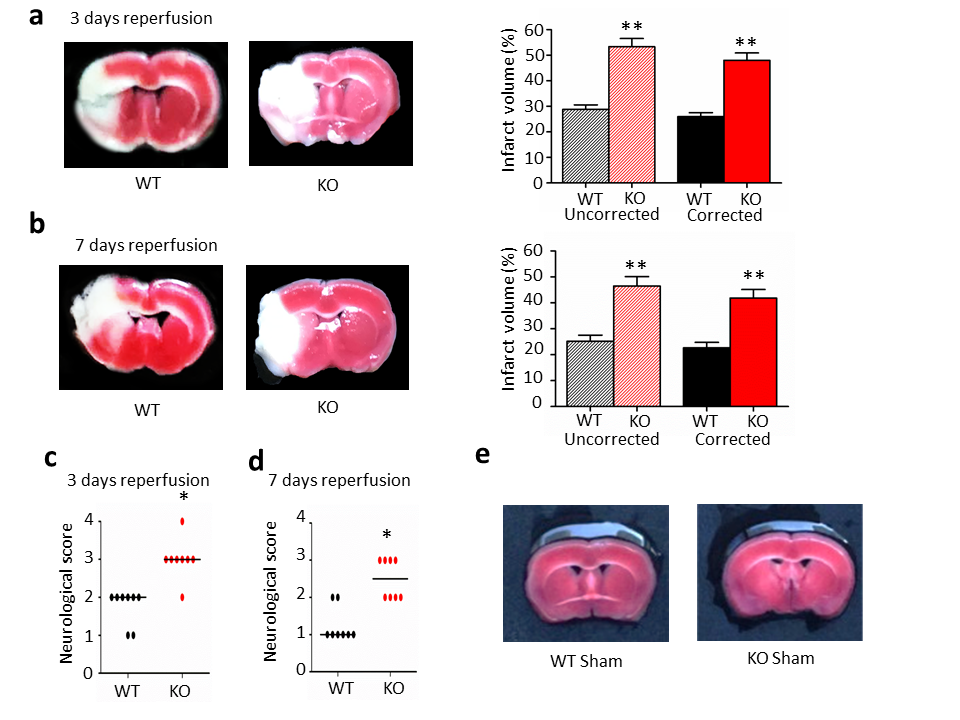


**Fig. S3** NDRG2 deficiency exacerbates focal cerebral ischemia. WT and KO mice were subjected to MCAO and 3-day (a) or 7-day (b) reperfusion (n = 8). Representative coronal brain sections stained for TTC quantification of the infarct volume and neurological scoring (c) and (d) after tMCAO. a, b, ^**^*p <* 0.01, Student’s *t*-test; c, d, ^*^*p <* 0.05; Wilcoxon rank sum test. (e) Representative coronal brain sections of WT and KO mice following a sham operation


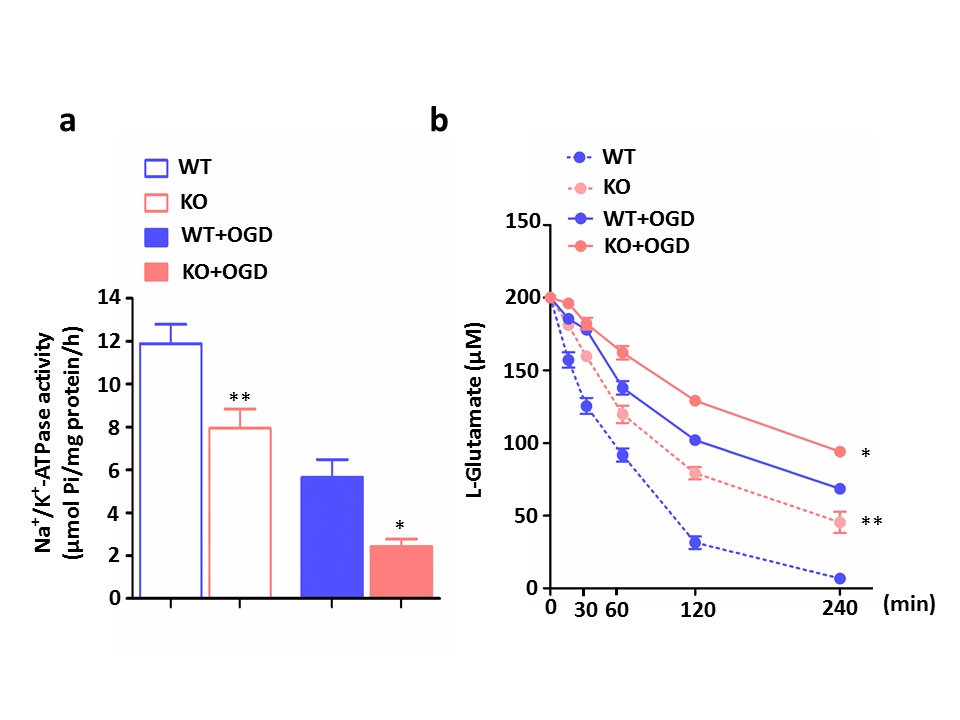


**Fig. S4** NDRG2 deficiency aggravates OGD-induced impairment of astrocytic Na^+^/K^+^-ATPase and glutamate uptake. (a) Na^+^/K^+^-ATPase activity was determined following the experimental procedure in WT and KO astrocytes with or without OGD treatment. Data are expressed as the means ± SEM from three independent determinations, each of which was performed in quadruplicate and evaluated using one-way ANOVA followed by Tukey-Kramer’s post hoc test. ^**^*p* < 0.01 versus WT, ^*^*p* < 0.05 versus WT + OGD. (b) Glutamate levels in the media of cultured WT and KO astrocytes with or without OGD treatment were measured at various time intervals. Data were obtained from three independent measurements, and each experiment was performed in quadruplicate. ^**^*p* < 0.01 versus WT, ^*^*p* < 0.05 versus WT + OGD; repeated-measures ANOVA


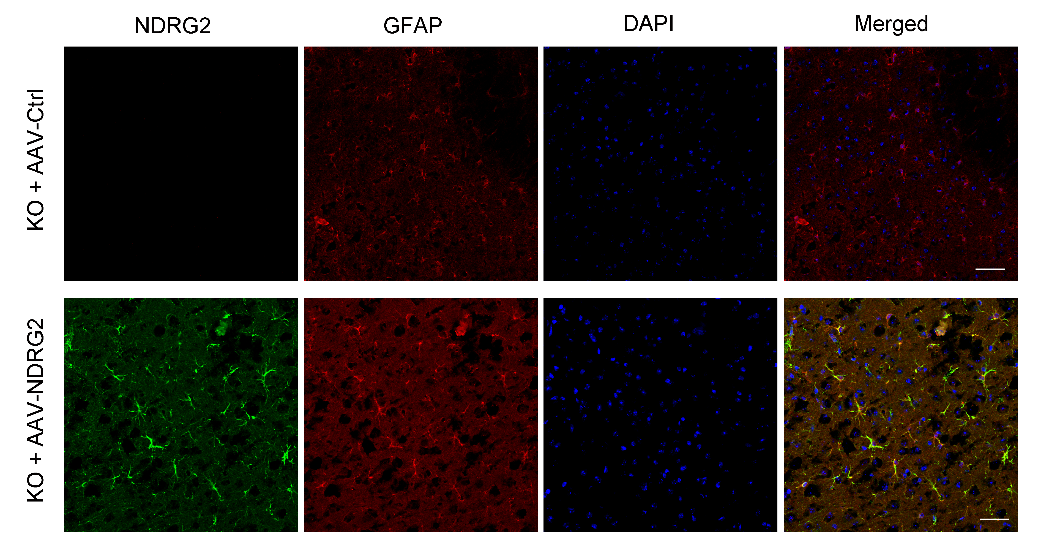


**Fig. S5** NDRG2 virus injection rescues NDRG2 expression in the astrocytes of *Ndrg2^-/-^* mice. Representative fluorescence images of NDRG2 (green), glial fibrillary acidic protein (GFAP, red), and DAPI (blue) staining in the striatal ischemic penumbra of *Ndrg2^-/-^* mice following an intracerebroventricular injection of the *NDRG2* virus (AAV-NDRG2) or a control virus (AAV-Ctrl). Merged images are shown. Scale bar = 50 µm


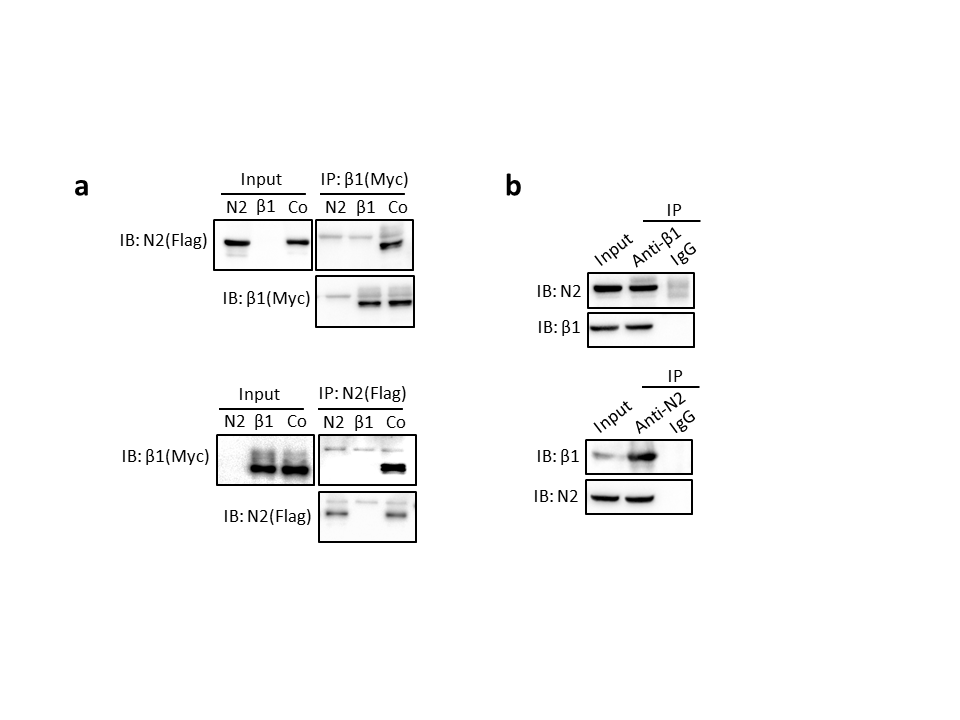


**Fig. S6** NDRG2 interacts with Na^+^/K^+^-ATPase β1. (a) HEK293 cells were transfected with a full-length plasmid encoding Flag-NDRG2 and/or full-length plasmid encoding Myc-Na^+^/K^+^-ATPase β1 for 48 hours, and the inputs and immunoprecipitates were subjected to IB analysis using an anti-Flag or anti-Myc antibody as indicated. Similar results were obtained in three separate experiments. Co: N2: NDRG2; β1: Na^+^/K^+^-ATPase β1; Cotransfection. (b) Interaction between endogenous NDRG2 and Na^+^/K^+^-ATPase β1 in primary cultured mouse astrocytes. Immunoprecipitation (IP) assays were performed using whole-cell lysates of astrocytes pretreated with protein A/G-conjugated sepharose beads. Whole cell lysates (Input) and immunoprecipitates were subjected to IB analysis using an anti-NDRG2 or anti-Na^+^/K^+^-ATPase β1 antibody


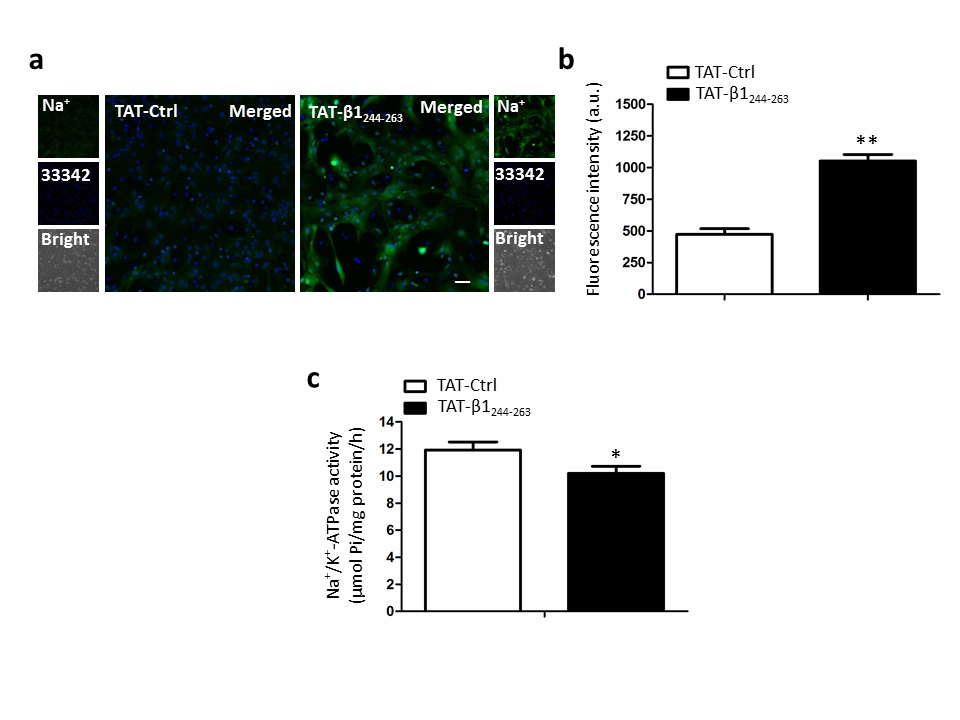


**Fig. S7** TAT-β1_244-263_ decreases the transmembrane Na^+^ gradient and Na^+^/K^+^-ATPase activity in astrocytes. (a-c) Cultured astrocytes were incubated with 20 μM TAT-β1_244-263_ or TAT-Ctrl for 2 hours. (a) Representative fluorescence images showing CoroNa Green trapped inside the cytoplasm of astrocytes, which were counterstained with Hoechst 33342. Scale bar = 20 μm. (b) Green [fluorescence](javascript:void(0);) [intensity](javascript:void(0);) of CoroNa in each group. Data are presented as the means ± SEM of three independent sets of cultures, and each group was imaged in 5 fields of view and analyzed using Student’s *t*-test. ^**^*p* < 0.01 versus the TAT-Ctrl. (c) Na^+^/K^+^-ATPase activity in astrocytes was measured as described in the Methods section. Data represent the means ± SEM of three independent determinations, each of which was performed in quadruplicate and analyzed using Student’s *t*-test. ^*^*p* < 0.05 versus TAT-Ctrl


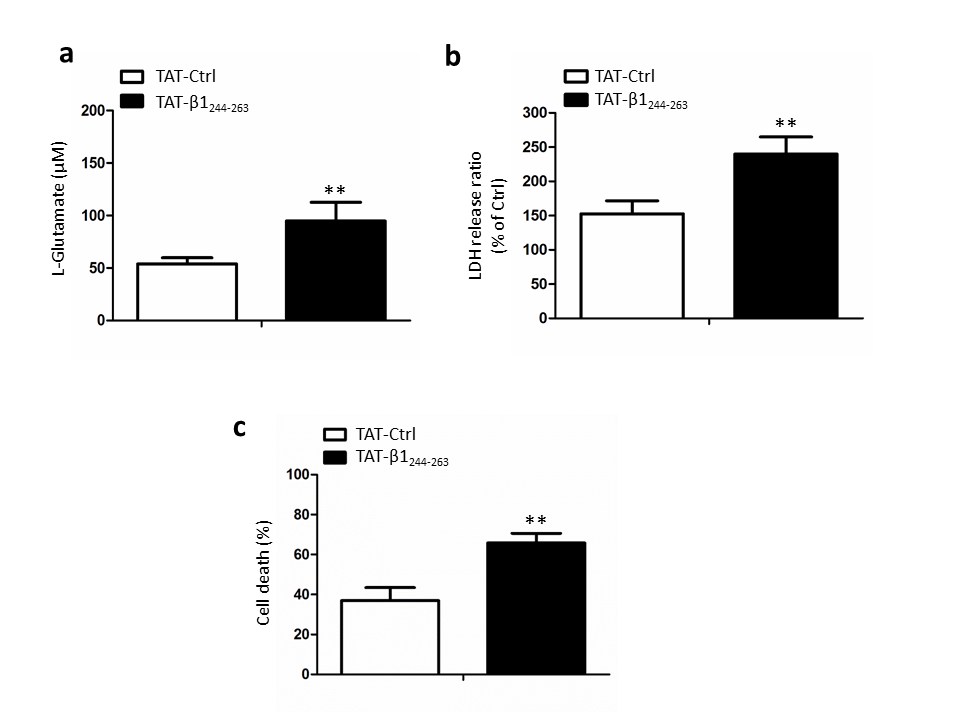


**Fig. S8** TAT-β1_244-263_ reduces astroglial glutamate clearance and increases neuronal death following a glutamate challenge *in vitro*. (a-c) Astrocytes were treated with TAT-β1_244-263_ or TAT-Ctrl for 2 hours and then indirectly cocultured with neurons. (a) The glutamate levels in the medium were measured after the cocultures were exposed to 200 μM glutamate for 2 hours. LDH release from neurons (b) and neuronal death (c) was detected 2 hours after the glutamate treatment and another 24 hours after further culture without glutamate. Data represent the means ± SEM of three independent determinations, each of which was performed in quadruplicate and analyzed using Student’s *t*-test. ^**^*p* < 0.01 versus TAT-Ctrl


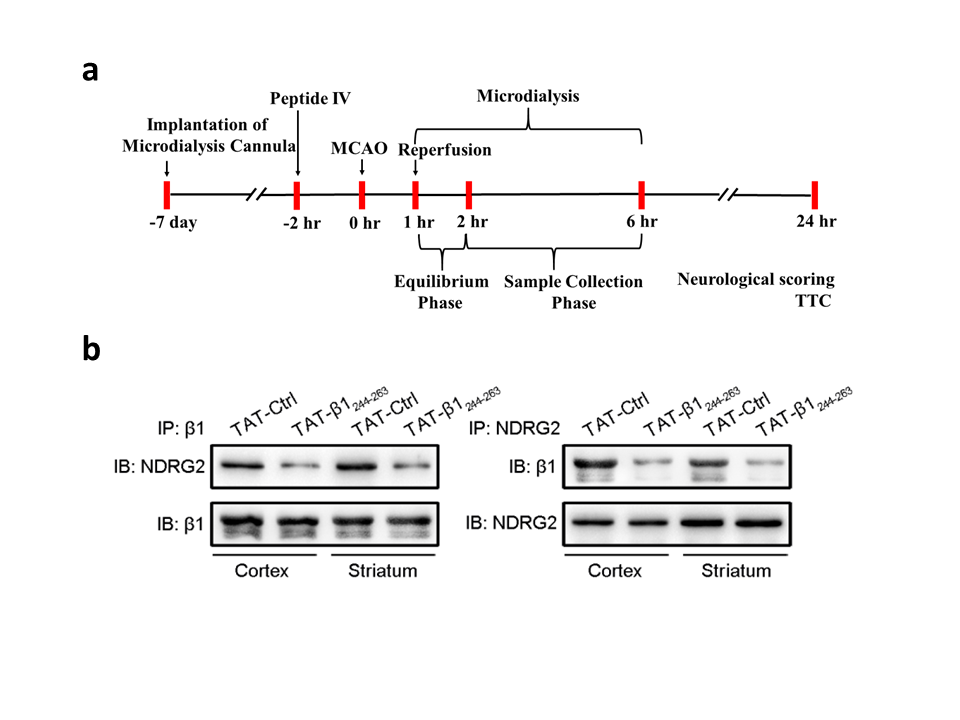


**Fig. S9** Dissociation of the NDRG2-Na^+^/K^+^-ATPase β1 interaction abolishes the NDRG2-mediated cerebral protection *in vivo*. (a) Diagram of the experimental procedure shown in Figure 4i-l. (b) Co-IP analysis of NDRG2 and Na^+^/K^+^-ATPase β1 using cortical and striatal protein samples from mice intravenously injected with TAT-Ctrl or TAT-β1_244-263_ (10 mg/kg). Similar results were observed in each of three independent experiments


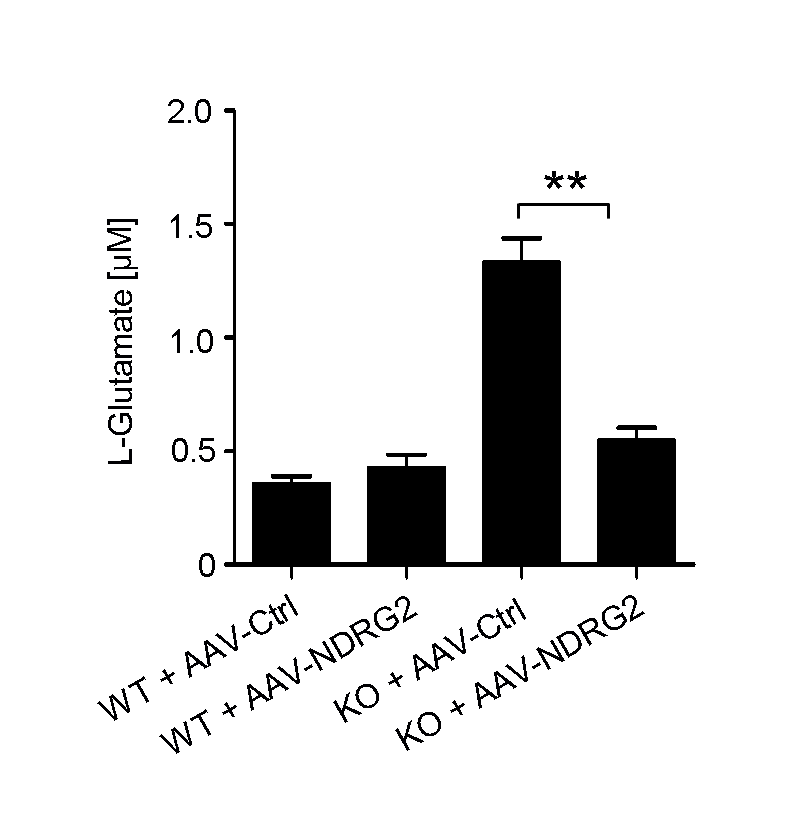


**Fig. S10** Microdialysis samples were collected from striatal extracellular fluid following a sham operation as indicated (n = 6). The concentrations of glutamate in the microdialysis samples were measured by HPLC. Data were evaluated using one-way ANOVA followed by Tukey-Kramer’s post hoc test. ^**^*p* < 0.01 versus KO + AAV-Ctrl. Error bars, mean ± SD

**Supplementary tables**

**Table S1.** Sources and dilutions of antibodies used for the IF and IB experiments.

| Antigen | Dilution (IF) | Dilution (IB) | Source |
| --- | --- | --- | --- |
| NDRG2 | 1:200 | 1:1000 | Abcam/CST |
| GFAP | 1:150 |  | CST |
| EAAT1 |  | 1:1000 | Abcam |
| EAAT2 |  | 1:1000 | Abcam |
| Na^+^/K^+^-ATPase α1 |  | 1:2000 | CST |
| Na^+^/K^+^-ATPase β1 |  | 1:1000 | Sigma |
| Flag-tag |  | 1:1000 | CST |
| Myc-tag |  | 1:1000 | CST |
| β-tubulin |  | 1:2000 | CST |

CST, Cell Signaling Technology; IB, immunoblotting; IF, immunofluorescence.

**Table S2.** Candidate positive clones of the NDRG2 interaction screened using a yeast two-hybrid system.

| Clone | Gene names |
| --- | --- |
| 13  73  109 | Na^+^/K^+^-ATPase β1 polypeptide (ATP1B1) |
| 31  79 | Cannabinoid receptor interacting protein 1 (CNRIP1) |
| 19 | SH3-domain GRB2-like interacting protein 1 (SGIP1) |
| 27 | Unc-84 homolog B (UNC84B) |
| 37 | Poly (rC) binding protein 1 (PCBP1) |
| 42 | Calpain 7 (CAPN7) |
| 76 | Procollagen C-endopeptidase enhancer (PCOLCE) |
| 106 | Lipoprotein lipase (LPL) |
| 115 | Chitinase domain containing 1 (CHID1) |
